# Development and verification of a novel immunogenic cell death‐related signature for predicting the prognosis and immune infiltration in triple‐negative breast cancer

**DOI:** 10.1002/cnr2.2007

**Published:** 2024-03-01

**Authors:** Jiachen Li, Zhengtian Li, Wenkang Yang, Jianmin Pan, Huazong You, Lixiang Yang, Xiaodong Zhang

**Affiliations:** ^1^ Department of Gastrointestinal and Gland Surgery The First Affiliated Hospital of Guangxi Medical University Nanning China; ^2^ Department of Bone and Joint Surgery The First Affiliated Hospital of Guangxi Medical University Nanning China

**Keywords:** immune infiltration, immunogenic cell death, immunotherapy, triple‐negative breast cancer, tumor microenvironment, tumor mutational burden

## Abstract

**Background:**

Insufficient understanding of the pathogenesis and tumor immunology of triple‐negative breast cancer (TNBC) has limited the development of immunotherapy. The importance of tumor microenvironment (TME) in immunotyping, prognostic assessment and immunotherapy efficacy of cancer has been emphasized, however, potential immunogenic cell death (ICD) related genes function in TME of TNBC has been rarely investigated.

**Aims:**

To initially explore the role and related mechanisms of ICD in TNBC, especially the role played in the TME of TNBC, and to identify different relevant subtypes based on ICD, and then develop an ICD‐related risk score to predict each TNBC patient TME status, prognosis and immunotherapy response.

**Methods and results:**

In this study, we identified distinct ICD‐related modification patterns based on 158 TNBC cases in the TCGA‐TNBC cohort. We then investigated the possible correlation between ICD‐related modification patterns and TME cell infiltration characteristics in TNBC. By using univariate Cox and least absolute shrinkage and selection operator (LASSO) regression analysis, we created a risk scoring system (ICD score) to quantifiably evaluate the impact of ICD‐related modification patterns in individual TNBC patient. Two different ICD‐related modification patterns were found with significant differences in immune infiltration. Lower ICD score was correlated with higher immune infiltration, tumor mutational burden and significantly enriched in immune‐related pathways, indicating a strong ability to activate immune response, which might account for relatively favorable prognosis of TNBC patients and could serve as a predictor to select suitable candidates for immunotherapy. We used two independent cohorts, GSE58812 cohort and Metabric cohort to validate prognosis and immunohistochemistry for preliminary in vitro validation.

**Conclusion:**

This study evidenced that the ICD‐related modification patterns might exert pivotal roles in the immune infiltration landscape of TNBC and ICD score might act as potential predictors of prognostic assessment and immunotherapy response. This research provides unique insights for individualize immune treatment strategies and promising immunotherapy candidates screening.

## 1 INTRODUCTION

Breast cancer has become one of the most concerned malignant tumors in women in recent years due to its increasing incidence.^1^ Triple‐negative breast cancer (TNBC) which is the most malignant form of this highly heterogeneous cancer has the following typical features: lack expression of estrogen receptor (ER), progesterone receptor (PR) and human epidermal growth factor receptor 2 (HER2),^2^ and accounts for about 12%–17% of cases.^3^ This type of breast cancer, whose 4‐year survival rate is only about 77%, usually holds the worse prognosis because of its high aggressiveness and heterogeneity.^2^, ^4^ Despite the use of nonspecific systemic chemotherapy and radiotherapy or some other methods, treatment benefits for patients with TNBC remain limited.^5^ It is essential to explore brand new treatment strategies against TNBC. Therefore, immunotherapy emerging in recent years may be a promising measure to treat TNBC patients.

Over the last few decades, increasing evidences suggest that numerous molecules can be used as targets for immunotherapy in TNBC, such as targeting immune checkpoints including programmed death‐1/programmed death ligand‐1 inhibitors, cytotoxic T‐lymphocyte associated antigen‐4 inhibitor, etc.^6^ Immunotherapy drugs such as monoclonal antibodies against the corresponding receptors have generated reliable efficacy in many tumors such as endometrial cancer, lung cancer, pancreatic cancer, kidney cancer, myeloma and so on.^7^, ^8^, ^9^, ^10^, ^11^ However, immunotherapy is not effective against all types of cancer. Studies have shown that just a little part of TNBC patients respond well to immunotherapy (PD1/PD‐L1).^12^ Therefore, accurately predicting the immunotherapy response of TNBC patients to screen out patients who are suitable for immunotherapy, and clarifying the underlying mechanisms causing different immunotherapy responses will be major issues to be solved in the future.

At the same time, scientists are working to study TNBC tumor subtypes, such as immune subtypes.^13^ According to the literature, different TNBC subtypes show completely different prognostic characteristics.^14^ The tumor microenvironment (TME) has played an increasingly important role in cancer prognosis in recent years.^15^ Different immune infiltration status in the TME can lead to very different prognosis of tumor patients.^16^, ^17^ What is more, studies have shown that TNBC with different immune status has different prognosis.^18^, ^19^ Therefore, we speculate that TNBC may have subtypes with different TME immune infiltration characteristics, and there may be differences in the response to immunotherapy among different TNBC subtypes.

Immunogenic cell death (ICD) is part of regulatory cell death with the ability to trigger adaptive immune responses.^20^, ^21^ Over the past few years, numerous studies have identified damage‐associated molecular patterns (DAMPs), including high mobility group protein B1 (HMGB1), ATP and calreticulin (CRT), are the main factors affecting the immunogenicity of ICD.^22^ An important strategy of immunotherapy is to use different factors to trigger the anticancer response from immune system to kill cancer cells. Numerous studies have highlighted the crucial effect of ICDs in triggering anticancer immune responses.^23^ Lau et al. suggested that the effectiveness of paclitaxel relied upon the activation of antitumor immunity through ICD in ovarian cancer.^24^ Wang et al. indicated that oncolytic newcastle disease virus provoked the expression of ICD markers in prostate cancer cells, while combined inhibition of other targets such as STAT3 can enhance anti‐tumor effects against prostate cancer.^25^ These above showed that ICD may have a close relationship with prognosis of various tumors and the response of patients to immunotherapy. However, systematic studies on the role of ICD in TNBC are still lacking.

Therefore, this study intends to preliminarily explore the role and related mechanisms of ICD in TNBC through bioinformatics methods, especially the role played in the immune microenvironment of TNBC. In addition, according to the above speculation, our main target is to recognize different TNBC subtypes based on ICD then develop an ICD‐related signature that predicts the status of TME, prognosis, and immunotherapy response in each individual patient of TNBC. This research will provide unique insights and perspectives on ICD in TNBC.

## 2 MATERIALS AND METHODS

### 2.1 Data acquirement and procession

Through mining the raw date from The Cancer Genome Atlas (TCGA) database (https://portal.gdc.cancer.gov/), which contained gene expression data, mutational data, corresponding clinical data and so on, we acquired a total of 158 TNBC tissue samples and 113 para‐cancerous tissue samples for further analysis. The samples in GSE58812 cohort with transcriptome RNA sequences and comprehensive clinical information of 107 TNBC patients from the Gene Expression Omnibus (https://www.ncbi.nlm.nih.gov/geo/, GEO) database were used as the external validation cohort. Metabric cohort containing comprehensive information including gene expression profiles and the related survival data of 299 TNBC samples were used as another external validation cohort, collected from the cbioportal (https://www.cbioportal.org/) database. The elimination of batch effects owing to nonbiological technical biases was performed by the “ComBat” algorithm of the sva package^26^ among distinct cohort. After comprehensive review and synthesis of a wide range of previous works in the literature, ICD‐related genes were collected for further exploration.^22^, ^27^, ^28^ For specific ICD genes, please refer to Table S1.

### 2.2 Unsupervised clustering analysis

Based on the expression pattern of ICD‐related genes, unsupervised clustering analysis was carried out on gene expression matrix of TNBC patients via the “ConsensusClusterPlus” R package, then several sub‐clusters of samples were obtained.^29^ Cumulative distribution function and consensus matrix were used to determine the optimal number of clusters. This process which bootstrapped clustering trees was repeated 1000 times to ensure the clustering was accurate and stable. The K value which represented the appropriate number of clusters was determined when the variance within each cluster was minimized and the variance between clusters was maximized. Obtained clusters were subjected to followed survival analysis using the Kaplan–Meier method to further analyze. We also conducted a difference analysis on the expression levels of the ICD regulatory genes among subclusters to investigate a possible heterogeneity in different clusters, which was presented in a heatmap.

### 2.3 Gene enrichment analysis between different clusters

Gene set variation analysis (GSVA) was performed on different subclusters applying R package ‘GSVA’ to further explore the heterogeneity within clusters and their biological significances. The gene sets and 50 HALLMARK pathways for GSVA analysis were downloaded from MSigDB database^30^ (http://software.broadinstitute.org/gsea/msigdb/index.jsp). Differences of immune cell content in TME of various subclusters were analyzed by single‐sample gene set enrichment analysis (ssGSEA) method to explore the immune cell infiltrations in each cluster.

### 2.4 ICD phenotype‐related differentially expressed genes (DEGs) identification between different clusters

“limma” R package was applied to screen DEGs between different ICD clusters (filter criteria: |LogFC| > 1; *p* < .05), we termed them ICD phenotype‐related DEGs. Subsequently, Gene Ontology (GO) enrichment analysis and Kyoto Encyclopedia of Genes and Genomes (KEGG) enrichment analysis were conducted in ICD phenotype‐related DEGs to investigate the possible roles those DEGs played in the mechanisms of TNBC pathogenesis.

### 2.5 Development and external validation of ICD‐related prognostic signature

The prognosis value of ICD phenotype‐related DEGs were then determined by univariate cox regression analysis. We performed least absolute shrinkage and selection operator (LASSO) Cox regression analysis on ICD phenotype‐related DEGs with prognosis value to construct the prognostic gene signature using the R package “glmnet”. In LASSO regression, a tenfold cross‐validation was used to optimize the lambda (λ) until it produced the minimum mean cross validation error during each bootstrap iteration.^31^ At this point, we obtained a comprehensive prognostic scoring system called ICD score. The ICD score formula was calculated as follows:

Risk score=∑Expgenei*β

In the formula, *β* represents the weight coefficient of each gene signatures in LASSO regression and *Expgene* is the expression of each gene signatures. Based on the median value, TCGA‐TNBC samples were categorized into high and low ICD score groups. After classifying TNBC samples, we performed survival analysis to compare the prognosis of patients with different ICD score. Outcomes of this analysis mainly reflected in overall survival (OS), disease‐specific survival (DSS), progression‐free interval (PFI), and disease‐free interval (DFI). Kaplan–Meier analysis was used to compare the survival curves of the two groups. A ROC curve for 3‐, 5‐, and 7‐year survival were drawn to estimate predicting performance. Principal component analysis (PCA) and T‐distributed neighbor embedding (T‐SNE) were performed for classification reliability judgment.^32^ Univariate and multivariate analyses were conducted to determine the independence of ICD score. The ICD score was independently validated in the GSE58812 cohort and Metabric cohort to further validate the predictive ability and reliability of the model. The *p* value less than .05 indicated a significant difference.

### 2.6 Correlation analysis between ICD score and related pathways

After mining Molecular Signature Database (MsigDB, http://www.gsea-msigdb.org/gsea/msigdb/human/collections.jsp#H) and obtaining 50 HALLMARK pathways, we performed GSVA enrichment analysis using the R package ‘GSVA’ to investigate the role of ICD score in TNBC from the perspective of pathway.^33^

### 2.7 Immune feature analysis

The R package ‘estimate’ was used to calculate the TME scores including immune score, stromal score, and estimated score of each patient in the TCGA‐TNBC cohort. To investigate the association between ICD score and the components of TME, we estimated TME scores of each sample mentioned above to assess the proportion of the corresponding component in the TME and performed correlation analysis using Spearman coefficients. After gaining TME‐related pathway, we calculated the pathway score of each sample^34^ and then performed correlation analyses in the TCGA‐TNBC cohort to investigate the association of ICD score and immune cell infiltration, immunomodulatory genes, MHC genes, and chemokine/chemokine receptors. The visualization of all heatmaps in this step was conducted using the ‘ggplot2’ R package.

### 2.8 Prediction and verification of immunotherapy response for ICD score

Tumor mutational burden (TMB) has been considered to be biomarkers for immune checkpoint inhibitors (ICIs) response due to its potential impact in tumor immunity and close correlation with longer OS after immunotherapy across multiple cancer types.^35^, ^36^ To further predictive the immunotherapy response of ICD score, we performed difference analysis and correlation analysis between TMB and ICD score. To further validate the predictive ability of ICD score for immunotherapy response, we accessed transcriptome dataset sequence and clinical outcomes information of immunotherapy‐treated patients by mining publicly available datasets. Data for the investigation of immunotherapy response prediction was derived from following one cohort: IMvigor210 cohort.^37^ IMvigor210 cohort (*N* = 348) (http://research-pub.gene.com/IMvigor210CoreBiologies) which contained detailed individual participant data of advanced uroepithelial carcinoma treated with Atezolizumab (PD‐L1 monoclonal antibody) including gene expression data, clinical information, and immune therapy response records was selected.

### 2.9 Drug sensitivity analysis

The R package “pRRophetic” was implemented to assess the half inhibitory concentration (IC50) of different chemotherapy drugs.^38^ The sensitivity of the two risk groups to chemotherapy was then forecasted for searching the potential candidate drugs.

### 2.10 Immunochemistry assay

We collected cancer and adjacent cancerous tissues from 8 patients with TNBC in the Department of Gastrointestinal Surgery at the First Affiliated Hospital of Guangxi Medical University from March 2021 to January 2023 for immunohistochemical analysis (IHC). All tissues embedded in paraffin were cut into 4 mm‐thick slices. After deparaffinization and hydration, the slices were incubated overnight at 4°C with primary antibodies, including mouse anti‐DDX58 monoclonal antibody (1:200, TA506141S; ORIGENE) and rabbit anti‐TREM1 polyclonal antibody (1:100, TA369422S; ORIGENE), followed by incubation with secondary antibodies conjugated with horseradish peroxidase. The slices were washed with distilled water, stained with hematoxylin, and dehydrated. The samples were photographed using a microscope (Nikon), and the images were analyzed using ImageJ FIJI v2.1.0. Five high‐power fields were randomly selected, and the scoring was performed using the method of intensity score × percentage. The staining intensity was graded as follows: 0 (no staining), 1 (light brown), 2 (brown), and 3 (dark brown). The percentage of positive cells was divided into four levels: 1 (<5%), 2 (5%–30%), 3 (31%–60%), and 4 (61%–100%). The scores were presented as the means ± standard deviations and images were analyzed using GraphPad Prism 8.0.1 software (GraphPad Software, Inc.). Differences between groups were analyzed by the Wilcoxon rank‐sum test and Spearman correlation tests. The *p* < .05 was considered to indicate a statistically significant difference. The experiments were performed in triplicate. We also used the Human Protein Atlas database to identify the protein expression of these two ICD‐related genes in TNBC patients by IHC.

### 2.11 Statistical analysis

The software R (R version 4.1.2; https://www.r-project.org/) was used for statistical tests and data visualization in this study. Unsupervised clustering analysis was conducted using “ConsensusClusterPlus” R package. “limma” R package was used to screen DEGs between clusters. R package “glmnet” was used to construct the gene signature. We applied time‐dependent ROC curve analysis through “survivalROC” R package to assess ability of prediction. Gene enrichment analysis was performed using the R package “GSVA” and “ssGSEA”. R package ‘estimate’ was used to calculate the TME scores. The “ggplot2” package was used in the analysis of the correlation between ICD score and immune features and the visualization of all heatmaps. The R package “pRRophetic” was implemented in drug sensitivity analysis. GO and KEGG enrichment analysis was performed using the “ClusterProfile” R package. The difference analyses of the overall survival were performed via Kaplan–Meier analyses and a Kaplan–Meier curve was generated. The significance of correlation was analyzed by the Spearman correlation test. GraphPad Prism 8.0.1 software (GraphPad Software, Inc.) was used in immunochemistry assay analyses with Wilcoxon rank‐sum test and Spearman correlation tests. Indication of *p*‐value summaries were as follow: *p < .05, **p < .01, ***p < .001, and ****p < .0001.

## 3 RESULTS

### 3.1 ICDs genetic variation landscape in TNBC

**FIGURE 1:**
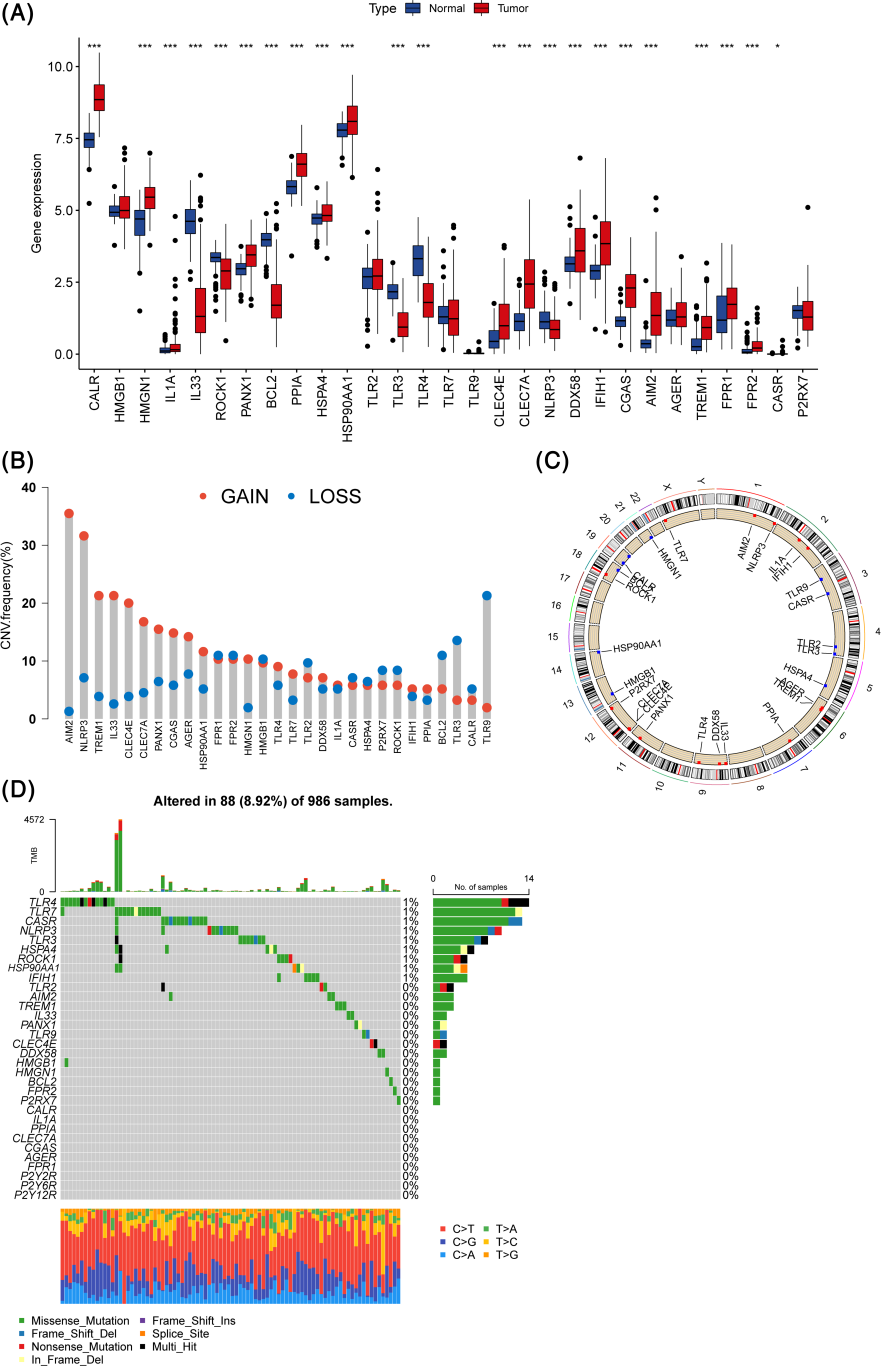
Landscape of genetic variation of the ICD‐related genes in the TNBC. (A) The difference of expression of ICD‐related genes in tumor and corresponding normal breast tissues. (B) ICD‐related genes variants frequency in the TCGA TNBC cohort. Red: amplification frequency. Blue: loss frequency. (C) Chromosomal mapping of mutations of the position of mutations of the ICD‐related genes on chromosome. (D) profiling of the somatic mutations of the ICD‐related genes in the TNBC.

The comprehensive information of 113 normal breast tissues and 158 TNBC tissues including RNA sequences and clinical date were downloaded from the TCGA database. After the acquirement of ICD‐related genes, we also compared the ICD‐related genes expression between paired normal breast tissue and TNBC tumor tissues with the results plotted in Figure 1A (*p* < .05). Copy number gains of ICD related genes were more frequent than copy number losses in TNBC, among which AIM2 and NLRP3 performed the most obviously (Figure 1B). We also plotted the CNV landscape of ICD‐related genes on human chromosomes in Figure 1C. As was shown in Figure 1D, overall mutation frequency of the ICD‐related genes in breast cancer were low.

### 3.2 Unsupervised clustering analysis

**FIGURE 2:**
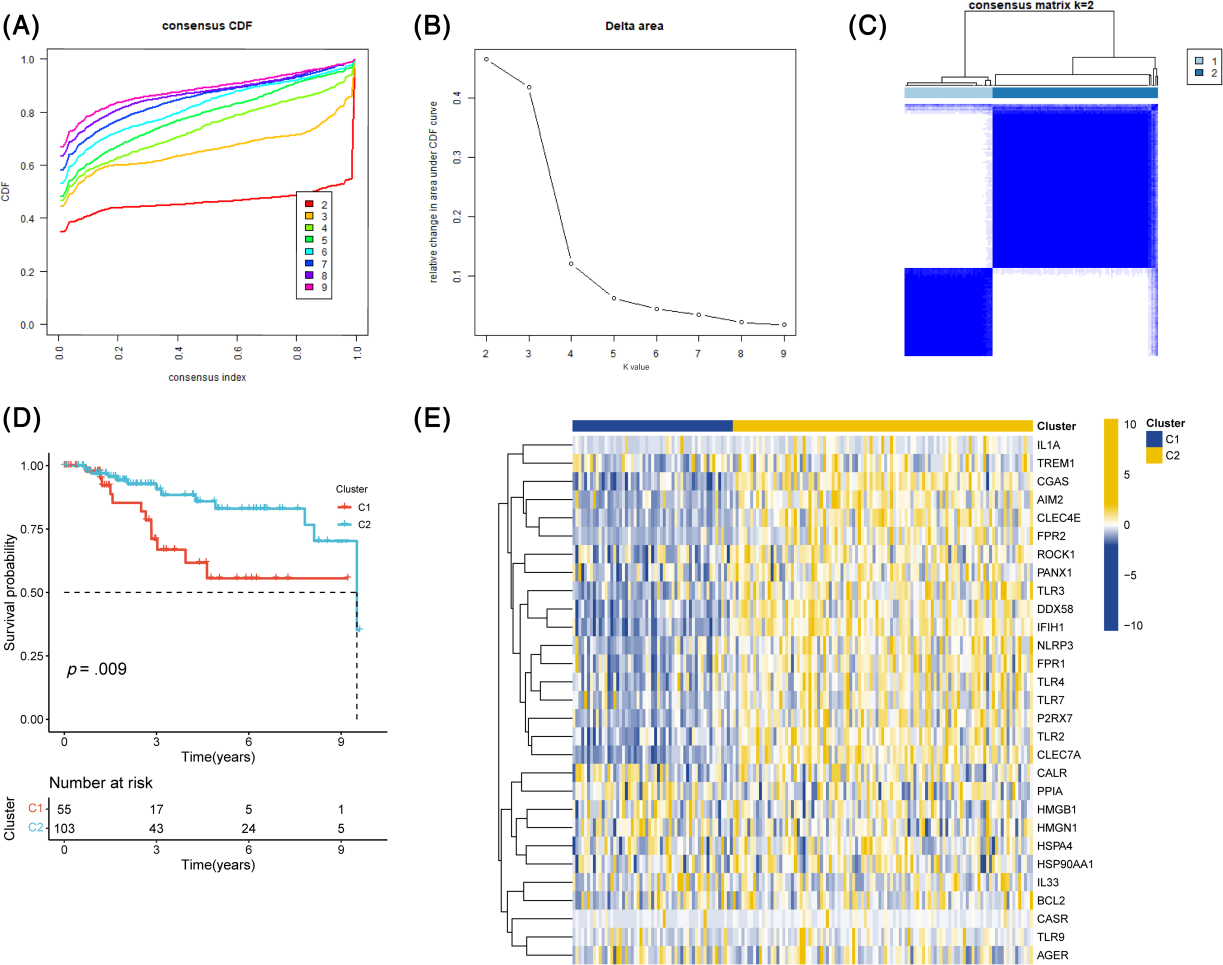
Cluster analysis for ICD‐related genes classification of the samples of TNBC, distinguishing 2 subgroups. (A) Cumulative distribution function curve, for determining the optimal number of clusters in consensus clustering. (B) Delta area curve of consensus clustering indicates the relative change in area under the cumulative distribution function (CDF) curve for k = 2 to 9. (C) Consensus matrix heat map defining 2 clusters of TNBC samples, corresponding to the consensus matrix for k = 2 obtained by applying consensus clustering. (D) Survival analysis of the 2 sub‐clusters. (E) Heatmap showing differential expression of ICD‐related genes across clusters.

We divided TNBC into distinct molecular subclusters through expression pattern of ICD‐related genes to better identify the roles of these genes in TNBC occurrence and progression. Defining the optimal number of clusters (K) as 2, the unsupervised clustering analysis produced two distinct clusters with minimal within‐group variance and maximum between‐group variance (Figure 2A–C). Significantly poorer prognosis of C1 were observed in subsequent survival analyses (Figure 2D) and attracted us to explore the heterogeneity between the two sub‐clusters. The heatmap in Figure 2E demonstrated the significantly differential expression of ICD genes in above two clusters.

### 3.3 GSVA and ssGSEA between different ICD molecular subclusters

**FIGURE 3:**
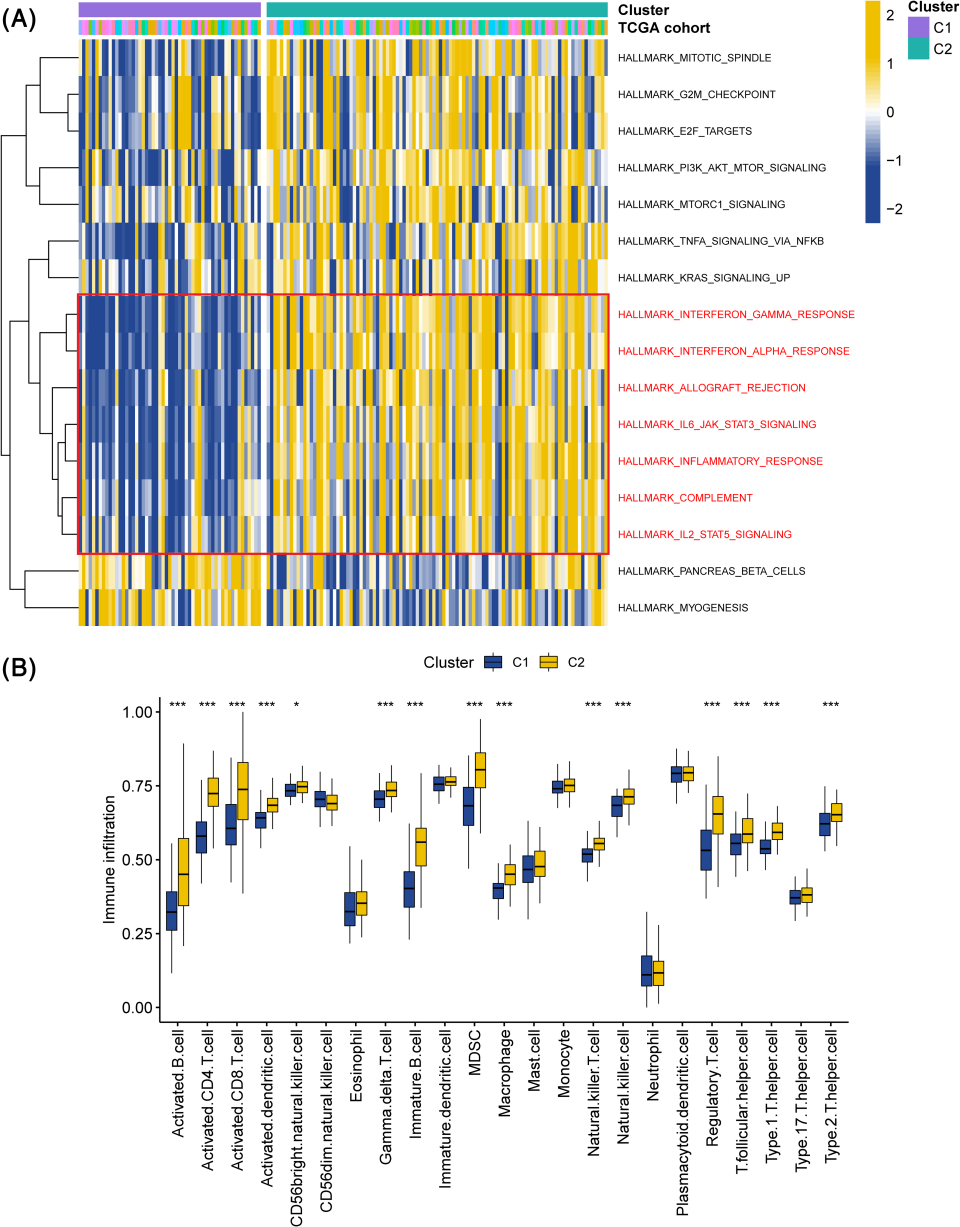
Difference analysis for the biological pathways and immune cell abundance across two clusters. (A) Heat map of GSVA cross two clusters, in which yellow and blue represent activated and inhibited pathways, respectively. Different colors in the TCGA cohort bands represent different individuals. (B) The comparison of immune cell types between 2 clusters.

Results of GSVA showed that cluster C2 mainly enriched in immune‐associated biological process, such as interferon gamma response, interferon alpha response, allograft rejection, IL6‐JAK‐STAT3 signaling, inflammatory response, complement and IL2‐STAT5 signaling (Figure 3A). After acquiring hallmark genes of 28 kinds of immune cells, we performed ssGSEA on samples from cluster C1 and C2 and calculated relevant enrichment score for each immune cell type in single TNBC sample. Then we analyzed the distribution of each immune cell type in cluster C1 and C2 to investigate the abundance differences of each infiltrating immunocyte in TME between cluster C1 and C2, which was shown in Figure 3B. Our finding suggested that the expression of ICD‐related genes may play a significant role in the tumor immunity and prompted us to focus on possible therapeutic application of ICD related indicators in immunotherapy for TNBC.

### 3.4 Gene enrichment analysis of ICD phenotype‐related DEGs

With the help of “limma” R package, we performed difference analysis among the two ICD clusters and identified 561 ICD phenotype‐related DEGs. Then, GO functional and KEGG pathway enrichment analysis was performed using the “ClusterProfile” R package, and the results were represented in Figure S1. The functional enrichment analysis revealed an enrichment of DEGs in functional categories related to immunity. Under biological process level, the DEGs were significantly enriched in terms of leukocyte mediated immunity and immune response‐regulating signaling pathway, etc. At the cell component level, DEGs were significantly enriched in external side of plasma membrane, T cell receptor complex, etc. The results under molecular function level showed that DEGs were mainly enriched in the antigen binding, cytokine receptor binding, immune receptor activity, etc (Figure S1A). The enrichment analysis of KEGG also indicated a significant enrichment of DEGs in pathways associated with immune responses, such as cytokine‐cytokine receptor interaction, cell adhesion molecules, chemokine signaling pathways, etc (Figure S1B). The above results suggest that DEGs associated with the ICD phenotype are involved in a variety of anti‐tumor immune‐related pathways.

### 3.5 Development and validation of ICD score in prognosis analysis

**FIGURE 4:**
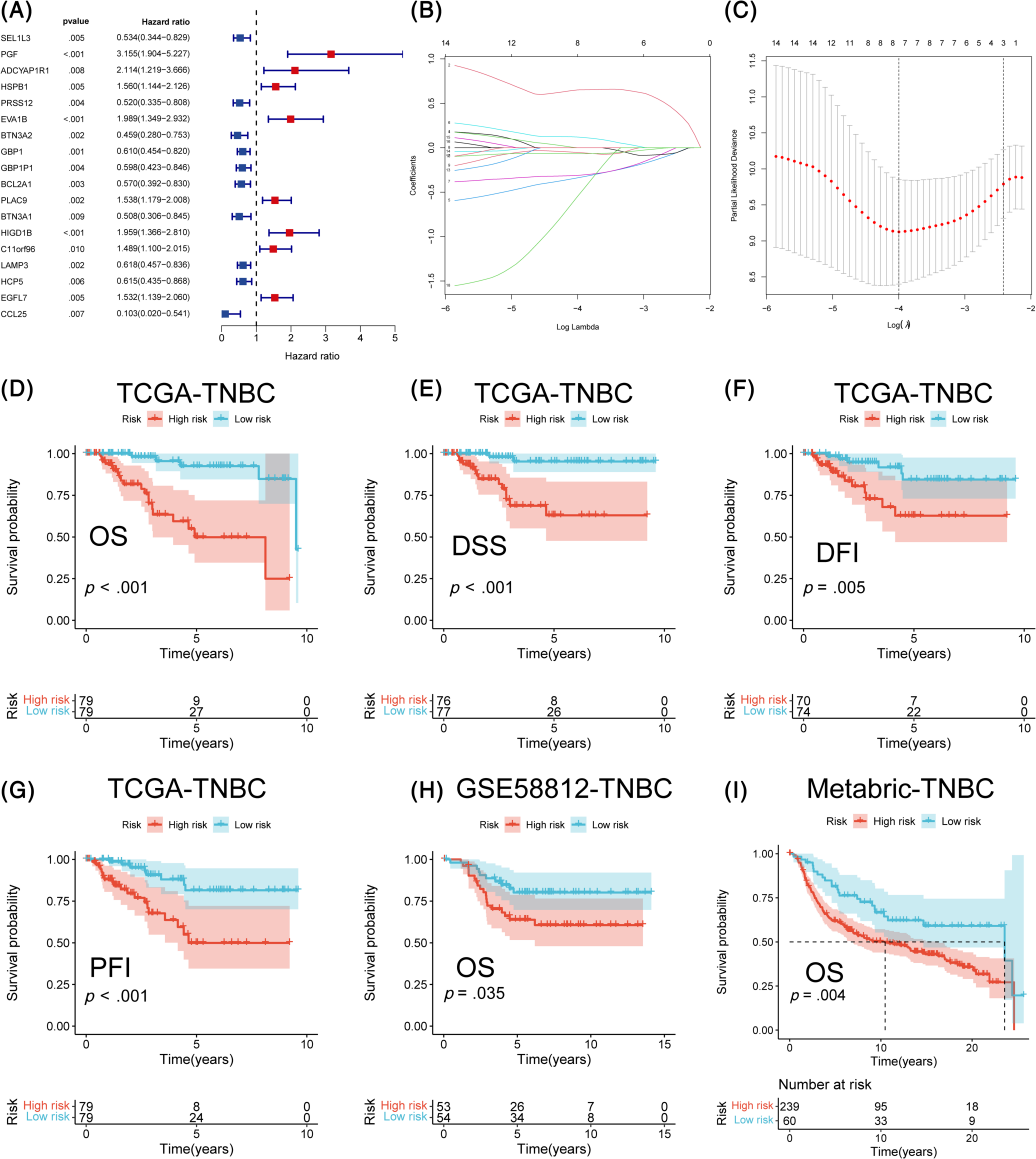
Construction of the prognosis ICD score model based on ICD‐related genes. (A) Forest plot for prognostic value analyses of ICD‐related gene signatures using univariate Cox regression model, where each curve corresponds to a single gene risk signature. (B) Factors selecting for Lasso regression Analysis. (C) The plot of regression coefficient diagram for Lasso regression Analysis, generated against the log (λ) sequence. (D–I) Kaplan–Meier survival analysis of the survival curves for the OS (D), DSS (E), DFI (F), PFI (G) of TNBC from TCGA database. Kaplan–Meier survival analysis of the overall survival curves for TNBC from GSE58812 dataset (H) and Metabric dataset (I).

We obtain 18 prognosis‐related genes by performing univariate Cox regression analysis to those ICD phenotype‐related DEGs (Figure 4A). Finally, we confirmed seven signature‐related genes by LASSO regression analysis with non‐zero coefficient (Figure 4B,C). The formula of ICD score was followed:

$$\begin{array}{l} \mathrm{ICD}\;\text{score}=\text{PGFexp.}\times 0.65117+\text{PRSS}12\exp.\\ \times \left(-0.33810\right)+\mathrm{EVA}1\text{Bexp.}\times 0.12054+\mathrm{BTN}3A2\exp.\\ \times \left(-0.31090\right)+\mathrm{GBP}1P1\exp.\times \left(-0.05320\right)+\mathrm{BCL}2A1\exp.\\ \times \left(-0.06643\right)+\mathrm{CCL}25\exp.\\ \times \left(-0.54437\right) \end{array}$$

**FIGURE 5:**
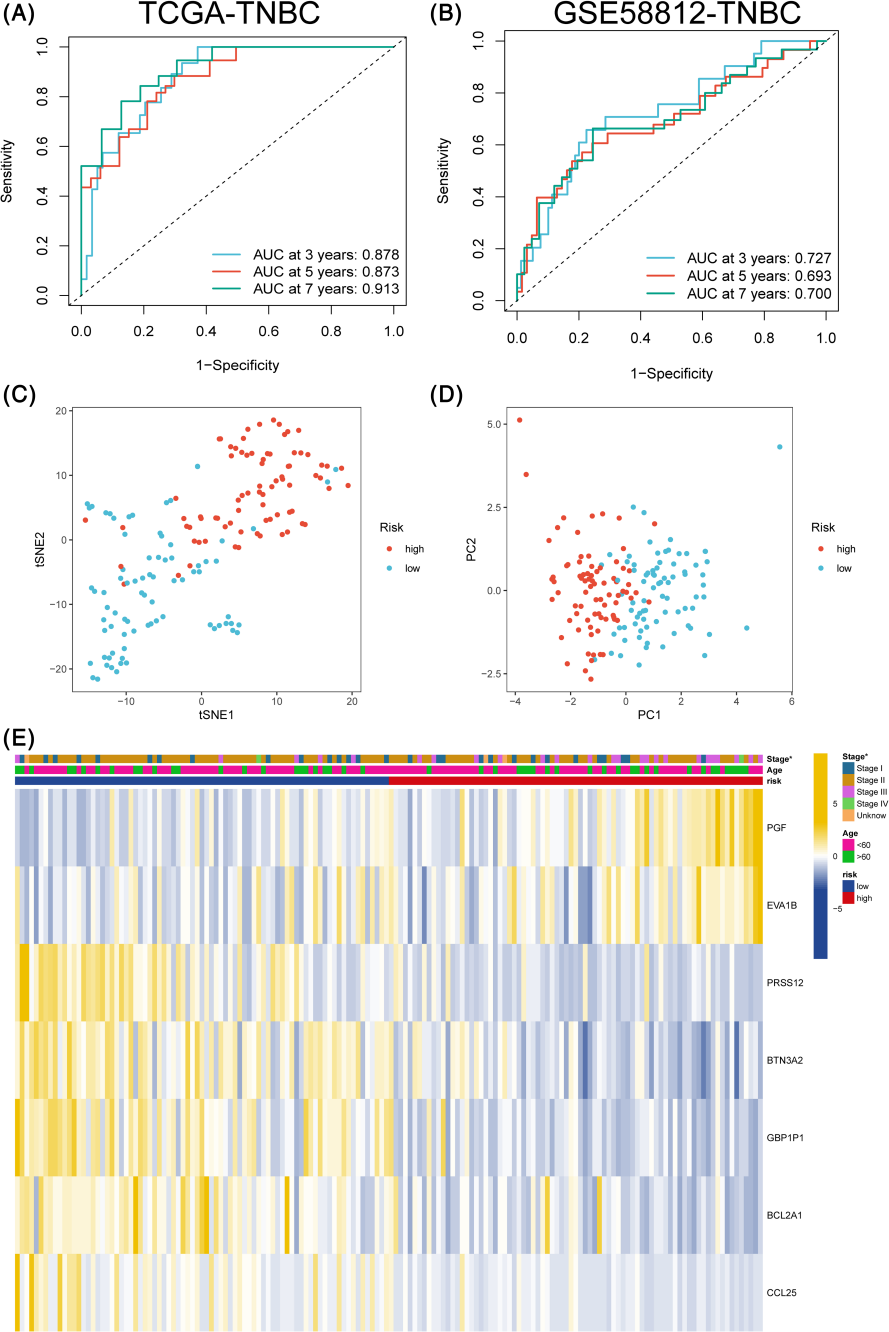
Evaluation of the prognosis ICD score model. (A, B) Time‐dependent ROC curve analysis of 3‐year, 5‐years, and 7‐years survival prediction for samples from TCGA and GSE58812. (C) tSNE plot revealing high and low risk group. (D) PCA plot of samples in high and low risk group. (E) Heatmap for the difference of main clinical characteristics, ICD score and 7‐gene signatures in high and low risk group.

**FIGURE 6:**
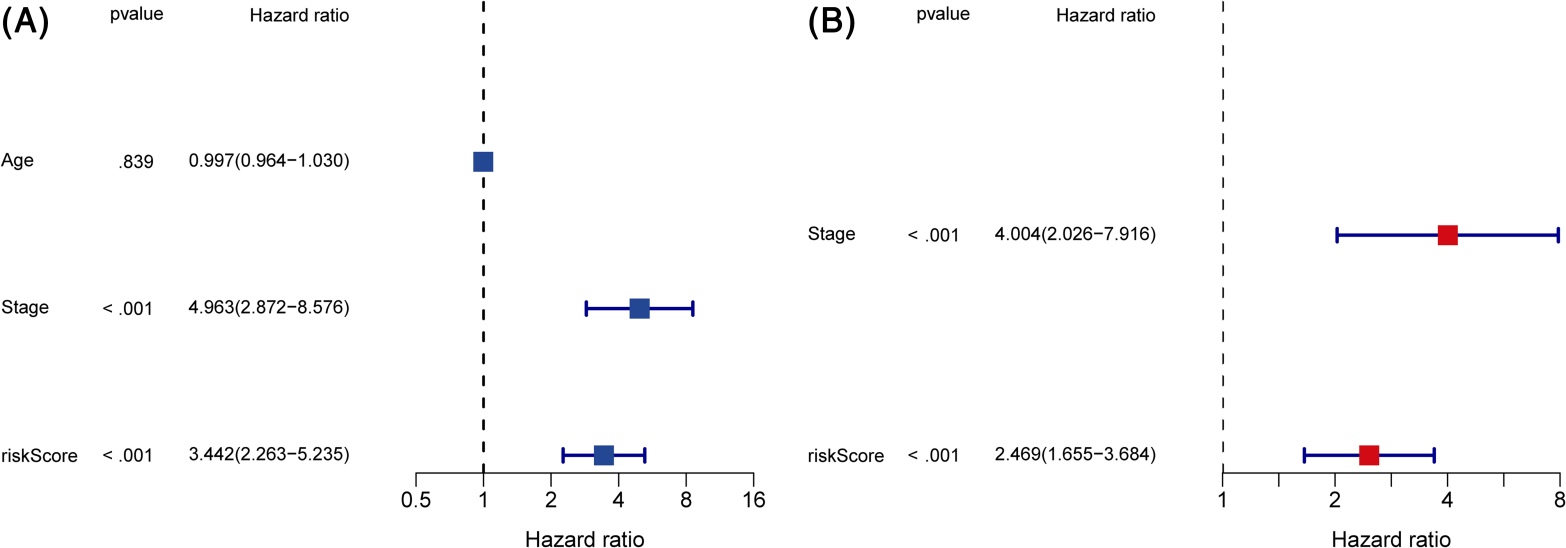
Prognosis value of main clinical characteristics and ICD score for TNBC. (A) Forest plot for Univariate analysis of prognostic factors. (B) Forest plot for Multivariate analysis of prognostic factors.

We divide TNBC samples into high and low ICD score groups by the median value. Next, we applied ICD score system to TNBC samples from TCGA and resultantly observed significant difference in OS, DFS, DFI and PFI between high and low risk groups (Figure 4D–G). Samples with lower ICD score tend to had more ideal prognosis. The OS can also be verified in the validation dataset of GSE58812 and Metabric cohorts (Figure 4H,I). ICD score also performed well in the prediction of 3‐, 5‐, 7‐years survival rates for TNBC patients from TCGA‐TNBC cohort (Figure 5A) and validating dataset of GSE58812 (Figure 5B). Additionally, based on the expression of the seven signature‐related genes, Results of PCA and t‐SNE analyses also showed good duplication within groups and significant differences between two sub‐groups, implying the ICD score system do well in the distinguishing of patients with different prognosis (Figure 5C,D). Results of difference analysis of the clinical features and signature‐related genes in the different risk groups were represented in a heatmap (Figure 5E). Results of univariate and multivariate Cox regression analysis indicated that ICD score had independence prognosis value for TNBC patients (Figure 6A,B). All the results above illustrated a significant link between ICD score and prognosis of TNBC patients and potential of ICD score as an independent prognostic indicator for TNBC.

### 3.6 GSVA of ICD score

**FIGURE 7:**
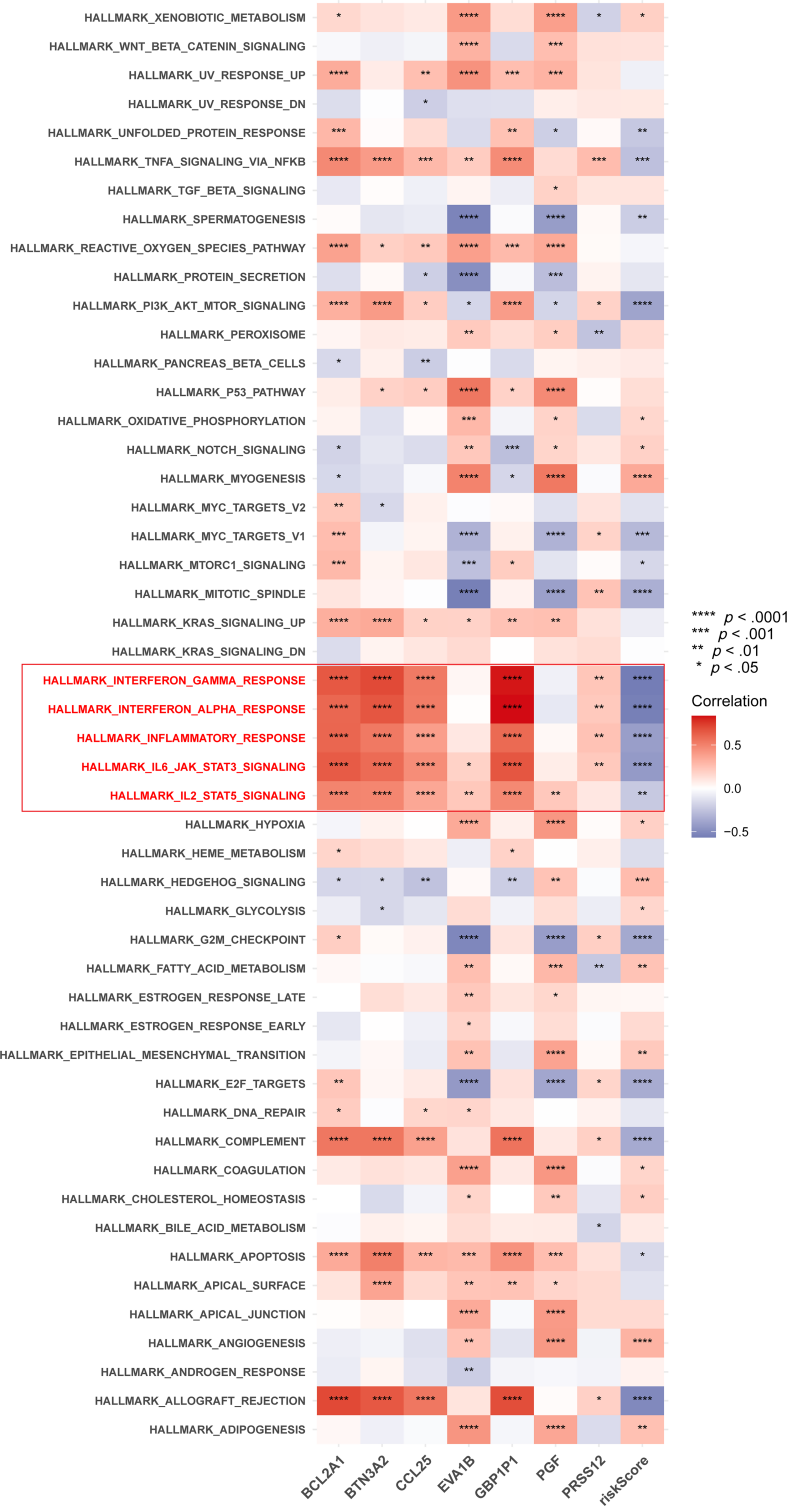
Correlation between biological function pathways and 7 hub genes as well as ICD score.

To further explore the biological impact of the ICD score, we collected 50 HALLMARK pathways and performed GSVA on ICD score. The correlation between genes and pathways we focused on were shown in Figure 7. We also reported negative correlations of ICD score and many immune‐related pathways including IL6‐JAK‐STAT3 signaling, IL2‐STAT5 signaling, interferon‐alpha response, interferon‐gamma response and inflammatory response. Notably, the pathways mentioned above all correlate with the tumor immune microenvironment.

### 3.7 Relationship between ICD score and the TME

**FIGURE 8:**
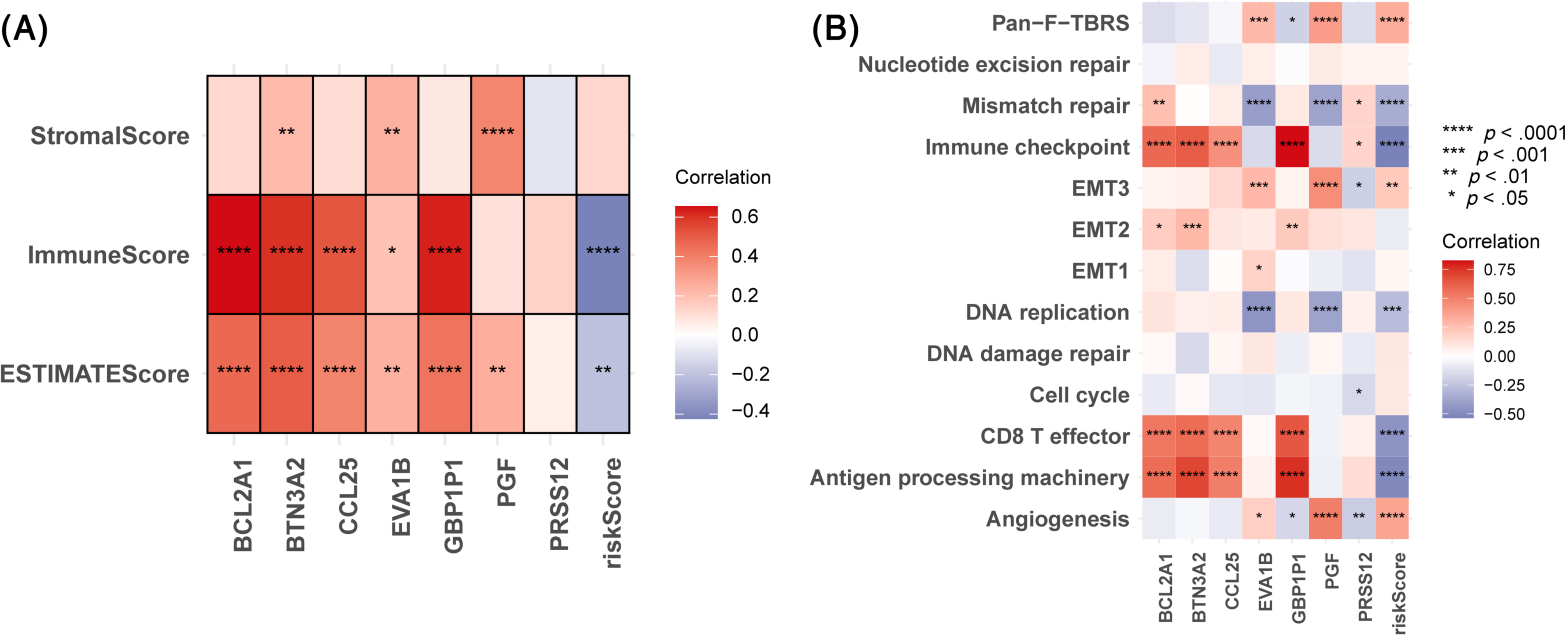
Correlation between TME and 7 hub genes as well as ICD score. (A) Correlation between immune score, stromal score, ESTIMATE score and 7 hub ICD genes as well as ICD score. (B) Correlation between TME‐related pathways and 7 hub genes as well as ICD score.

Our gaze shifted to the correlation between ICD score and TME. Results showed an inverse correlation between ICD score and Immune score, ESTIMATE score (Figure 8A). Results of followed analysis containing TME‐related pathways and ICD score together with the 7 hub ICD genes showed that ICD score was also negatively correlated with immune‐related pathways, including immune checkpoint, CD8^+^ T effector, and antigen processing machinery, whereas was positively associated with malignant‐promoting‐related pathways, including angiogenesis and pan‐F‐TBRS (Figure 8B). Both two analyses were similar. We presumed that in the TME, ICD score might reflect the suppress of anti‐tumor immunity and promotion of tumor cell proliferation and invasion.

### 3.8 Immune infiltrating analysis

**FIGURE 9:**
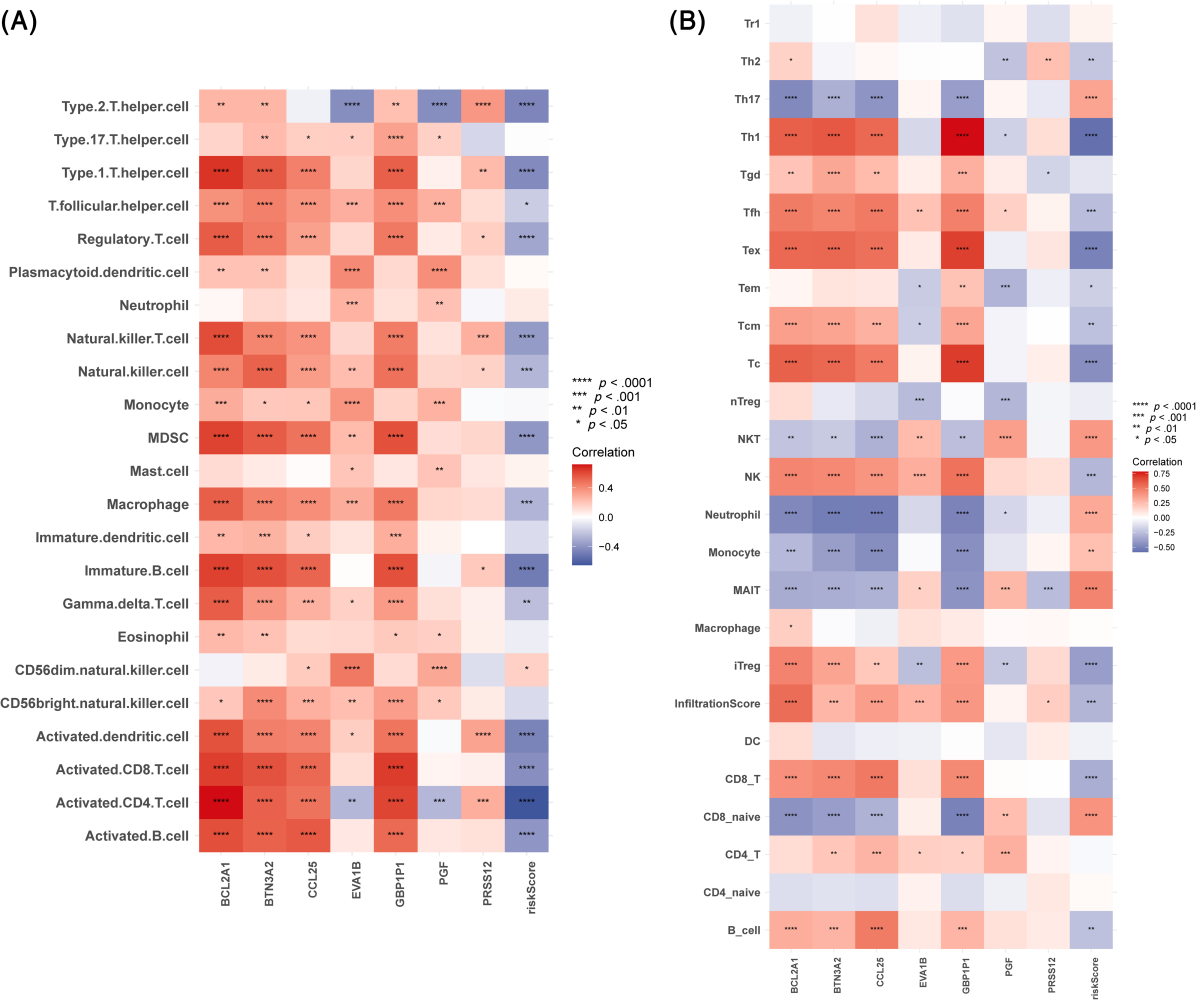
Immune cell infiltration analysis of 7 hub genes and ICD score. (A) Infiltration analysis of immune cells using ssGSEA. (B) Infiltration analysis of immune cells based on the ImmuCellAI database.

**FIGURE 10:**
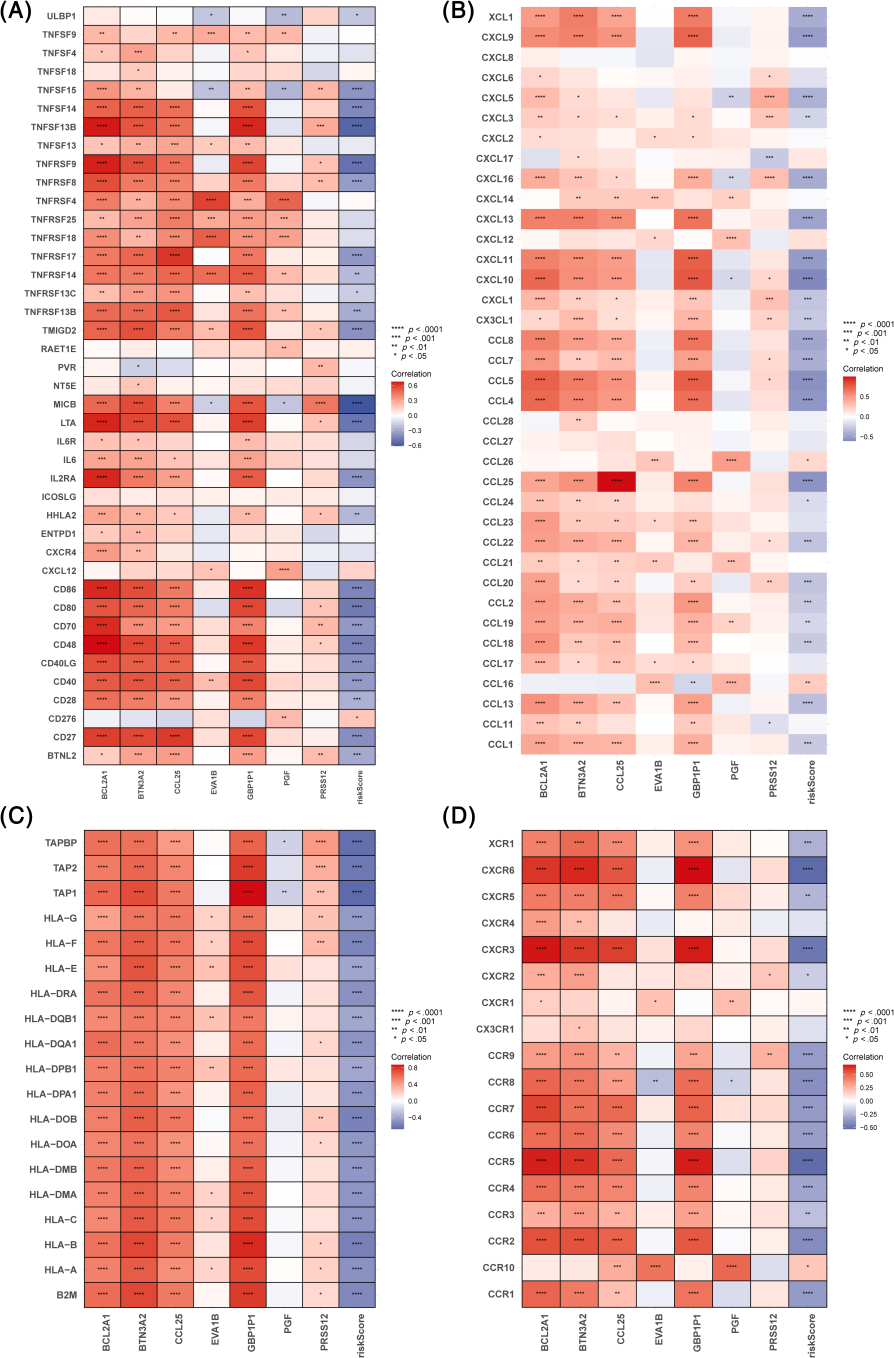
Correlation analysis of 7 hub genes and ICD score with immune cell markers. (A) Correlation of 7 hub genes and ICD score with immunomodulatory genes. (B) Correlation of 7 hub genes and ICD score with chemokines. (C) Correlation of 7 hub genes and ICD score with MHC genes. (D) Correlation of 7 hub genes and ICD score with chemokine receptors.

As the active component of TME, immune cells are vital in microenvironment and reflect the status of the immune microenvironment to some extent. We found that ICD score was negatively correlated with immune‐activated TME by performing infiltration analysis of immune cells using ssGSEA (Figure 9A). Lower ICD scores corresponded to more immune cell infiltration and stronger anti‐cancer immunity. Based on the ImmuCellAI database (http://bioinfo.life.hust.edu.cn/ImmuCellAI#!/), we found that the ICD score is negatively correlated with most immune T cells, indicating an immune‐suppressed TME (Figure 9B). These results again evidenced that high ICD scores represented an immunosuppressive microenvironment and poor prognosis. Further analyses indicated a significant negative correlation of ICD score with immune‐activating genes (Figure 10A), chemokines (Figure 10B), MHC genes (Figure 10C), and chemokine receptors (Figure 10D) in TNBC. In other words, TME patients with low ICD score seems to have abundant immune cell infiltration which mediates more active cellular immune response, thus stand for better prognosis.

### 3.9 The association between ICD score and immunotherapy response

**FIGURE 11:**
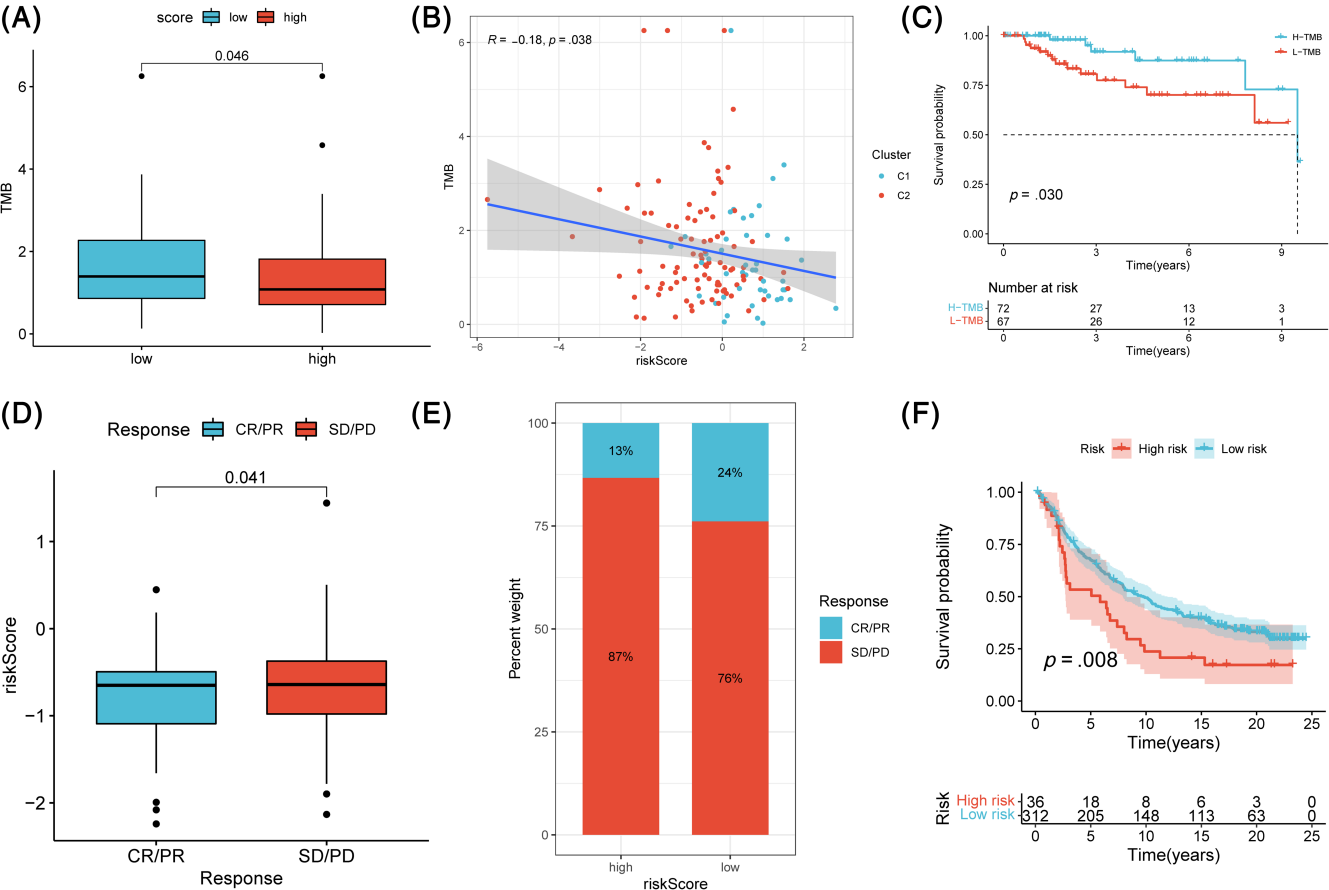
The correlation between ICD score and immunotherapy response. (A) TMB distribution across high and low risk group. (B) Correlation between TMB and ICD score in patients with TNBC. (C) Survival analysis for the overall survival of the high‐TMB group and low‐TMB group. (D) ICD score of patients with different therapeutic responses to immunotherapy. (E) Proportion of patients with different therapeutic responses to immunotherapy between high and low ICD score groups (CR/PR: complete response/partial response. SD/PD: stable disease/progressive disease). (F) Survival analysis for the overall survival of the high and low ICD score group.

Previous literature suggested that patients with a high TMB may be sensitive to ICIs treatment.^39^ A higher TMB was observed in the low‐ICD score group compared with the high‐ICD score group (Figure 11A) and further analysis confirmed that this relationship was negative (Figure 11B). We calculated the TMB for each TNBC samples from TCGA and divided them into high and low TMB groups by the optimal cutoff value. In the followed survival analysis, we found that patients in high TMB group had significantly better prognosis than patients in low TMB group (Figure 11C). From the aforementioned results, the following hypothesis can be proposed that lower ICD score implied a better immunotherapy response. We obtained immunotherapy samples (IMvigor210 cohort, *N* = 348) and calculated the ICD score of each sample to test this assumption. Results of survival analysis revealed a significantly more favorable survival of patients receiving immunotherapy with lower ICD score (Figure 11D). The objective response rate also exhibited significant difference between low ICD score group (24%) and high ICD score group (13%) and CR/PR group had a lower ICD score compared with the SD/PD group (Figure 11E). Survival analysis for the overall survival between the high and low ICD score group also showed the significant difference (Figure 11F). Our founding suggested that ICD score owned the potential to be an effective tool predicting response to immunotherapy.

### 3.10 The association between ICD score and drug sensitivity

**FIGURE 12:**
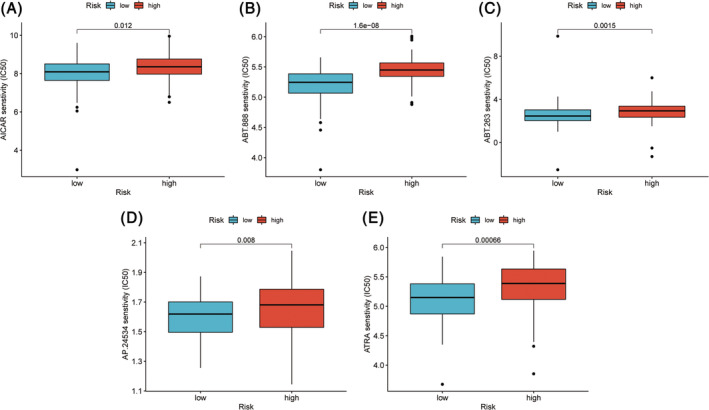
Drug sensibility analysis of ICD score. Differences in the IC50 of five drugs Acadesine (A), Veliparib (B), Navitoclax (C), Ponatinib (D), ATRA (E) in the high and low ICD score groups.

Beyond immunotherapy response, we parallelly performed drug sensitivity analyses on the response to chemotherapeutic agents and targeted therapeutics agents among TNBC patients with difference ICD scores. We found that IC50 values of five chemotherapeutics including Acadesine, Veliparib, Navitoclax, Ponatinib and ATRA, of samples in high‐risk group were higher compared with those in low‐risk group (*p* < .05), implying that patients with lower ICD score had more optimal treatment response to those medicine (Figure 12A–E).

### 3.11 Immunochemistry assay

**FIGURE 13:**
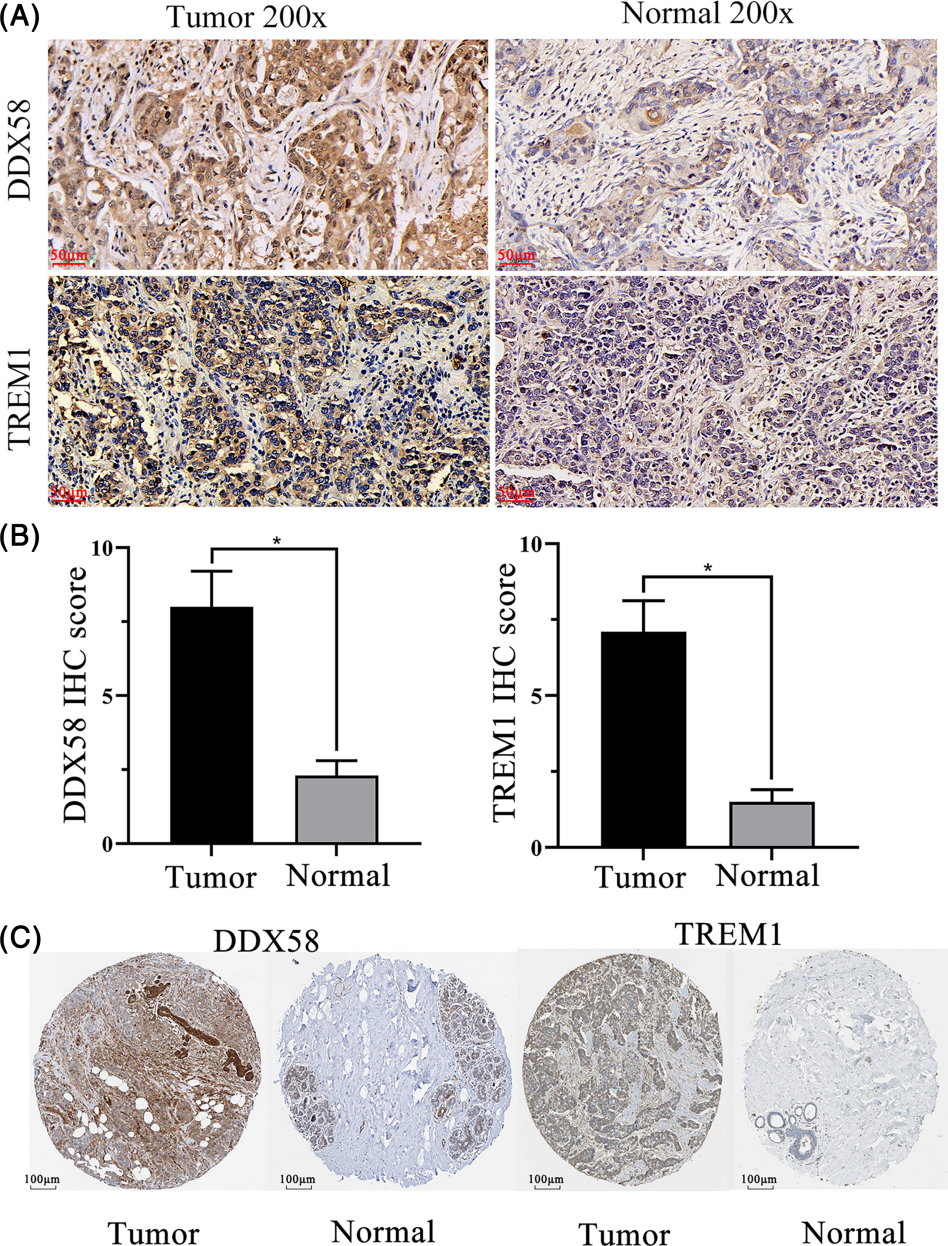
Immunohistochemical analysis of ICD‐related genes. (A) The representative images of IHC staining for DDX58 and TREM1 in TNBC and adjacent non‐tumor tissues (200×). (B) Quantification of A. *p < .05. (C) The representative images of IHC staining for DDX58 and TREM1 from Human Protein Atlas.

DDX58, also known as RIG‐I (Retinoic acid Inducible Gene I), is an important immune receptor that plays a crucial role in the process of viral infection. It can recognize RNA viruses such as influenza virus, hepatitis A virus, and hepatitis B virus, and initiate an immune response to resist viral infection. DDX58(RIG‐I) has an important role in the immune system and is of great significance in the fight against viral infections. TREM1 is a membrane receptor of the immunoglobulin superfamily, primarily expressed on myeloid cells such as macrophages and dendritic cells. Its function is crucial in inflammatory diseases such as infection, inflammatory bowel disease, arthritis, pneumonia, sepsis, and cancer. TREM1 can enhance the production of inflammatory mediators and cell infiltration, thereby strengthening the inflammatory response, and it is currently a hot topic of research in the field of immunotherapy. To further verify the reliability of our results, we selected two ICD‐related genes, DDX58 and TREM1, and collected cancer and adjacent non‐cancerous tissue specimens from 8 TNBC samples to detect the expression levels of DDX58 and TREM1 by IHC. Representative images of the IHC staining for DDX58 and TREM1 are shown in Figure 13A. We found that the IHC scores of DDX58 and TREM1 were higher in cancer tissues than in adjacent non‐cancerous tissues and the difference was significant (Figure 13B). We also obtained IHC staining images of DDX58 and TREM1 from the Human Protein Atlas database (Figure 13C), which once again verified the reliability of this study.

## 4 DISCUSSION

TNBC has attracted the attention of most researchers due to its unique molecular profile with lower 5‐year survival rates than other types of breast cancer patients.^4^ To make matters worse, immune therapies for TNBC are lacking. Therefore, we urgently need to explore novel immunotherapy‐related biomarkers with high accuracy which might improve patients' survival rate.

The ability of ICD to trigger antigen‐specific adaptive immune responses makes the field emerging at the forefront of cancer biology research, particularly in tumor progression and termination.^27^, ^40^ Mechanistically, dying cells signal the body to establish an immunological correlation between cellular stress and death. Healthy cells can drive inflammation but are insufficient to activate adaptive immunity. On the contrary, sufficient antigenicity can be expressed upon infection or malignancy or other external physicochemical influences, at which point cell death may lead to adaptive immune responses.^41^ Based on this, ICD can be used by us in the field of anti‐tumor research. Increasing evidences have proved that ICD not only plays a key role in inhibiting primary tumors but also in exerting abscopal effects and drastically suppressing distant or metastatic tumors.^42^, ^43^ Similar antitumor effects were also found in TNBC. Tukaramrao et al. found a new chemical compound called TPH104 to induce ICD in TNBC cell line through enlarging the stimulatory capacity of dendritic cells.^44^ Yasothhamani et al. designed a novel combined nanotherapeutic approach to incinerate the tumor under near‐infrared (NIR)‐triggered photothermal ablation and activate ICD, which exhibited powerful anticancer effects and effectively prevented tumor recurrence and metastasis in mouse models.^45^ Nowadays, there are not enough studies combining immunotherapy with ICD subtypes of TNBC, suggesting that there is still a huge space for ICD to explore in TNBC to prolong survival in patients with metastatic disease.

Meanwhile, in recent years, classifying tumors into more detailed subtypes has received more attention from scholars. Different types have their own applicable conditions and the common goal is to select appropriate patients from an extremely heterogeneous population to receive the corresponding treatment.^46^, ^47^ It is not uncommon for studies to attempt to subclassify TNBC further, since so‐called TNBC is a simple classification based on molecular receptors, which still contains numerous subsets with very different prognosis. Lehmann et al. used cluster analysis to identify 6 TNBC subtypes, each of which is suitable for different chemotherapy or targeted drugs with different prognosis.^48^ Although these tumor subtype classification methods can distinguish patients with different prognosis to a certain extent, they are not very good in classifying TNBC from the perspective of immune infiltration, which happens to be very important because immunotherapy will be the main method of TNBC treatment in the future. In our research, we filtered out ICD‐related genes most of which had significantly different expression between normal breast tissues and tumors. In order to identify the relevant TNBC types, we used these genes for typing in TCGA‐TNBC cohort. The survival probability of the two clusters was significantly different, and C2 had a clear survival advantage. In order to explore the possible mechanisms that may cause the different prognosis, we performed GSVA analysis showing that C2 was more enriched in immune activation‐related pathways including interferon response, allograft rejection, complement, indicating that good prognosis of C2 may be related to immune cell infiltration. Therefore, ssGSEA was performed and results also showed C2 had the highest score in almost all immune cells, which again suggested that the different prognosis of different types may be caused by different immune infiltration characteristics. The C2 may correspond to the immune inflamed phenotype (hot tumor) while C1 may correspond to the immune desert phenotypes (cold tumor).

Although our findings clearly defined the role of ICD in patient prognosis and the regulation of immune infiltration, the results were only derived from the large sample size population and cannot concretize ICD patterns in TNBC patients to the individual level. Considering the large individual heterogeneity in TNBC,^49^, ^50^ we have developed a scoring scheme defined as ICD score to quantify the ICD pattern in individual patients. This process was achieved by analyzing the identified ICD‐related signature genes. In detail, we took the differential genes between the two clusters and named them as ICD phenotype‐related DEGs. 7 genes were finally filtered out using univariate analysis and LASSO regression analysis. After modeling with the formula, patients were divided into high‐ and low‐risk groups taking the median as the bound, and results showed that high‐risk group had a worse prognosis for OS. Both external validation cohorts confirmed this again. At the same time, the scores also highly consistent with survival outcomes such as DSS, DFI, and PFI, indicating that the score was highly stable. The ROC curve also showed that this ICD score has reliable prognostic accuracy in the TCGA‐TNBC and GSE58812 cohort, respectively. Multivariate Cox regression analysis identified ICD score as an independent prognostic indicator.

Next, we explore the relationship between ICD score and immunity in more depth. First, GSVA showed that ICD score was inversely associated with immune‐related common star pathways such as IL6‐JAK‐STAT3 signaling, IL2‐STAT5 signaling, interferon‐alpha response, interferon‐gamma response and inflammatory response. This prompted us to further probe the role of ICD score in TME. Further results showed ICD score was negatively correlated with the immune score in the TME. To confirm this, we conduct a correlation analysis based on TME‐related pathways provided by a literature and the results strongly support our point. The results also showed that the ICD score had a negative relationship with immune activation‐related pathways but a positive relationship with those of tumor formation and progression such as angiogenesis and EMT3. Remarkably, the ICD score correlates positively with EMT3 but not with EMT2 and EMT1. This discrepancy arises from the distinct roles of each EMT type: EMT1 is linked to developmental processes like embryo formation and organ development, EMT2 is tied to repair processes such as wound healing and tissue regeneration, while EMT3 specifically relates to tumor metastasis.^51^ These all suggested that high ICD score corresponded to poor prognosis, possibly due to immunosuppression.

To gain a thorough comprehension of relationship between ICD score and specific components in the TME, we performed an immune cell infiltration correlation analysis on the ICD score by 2 well‐established methods. At the cellular level, both methods showed that the ICD score was inversely correlated with the majority of immune cells that matters in immunity. From the level of immune‐related molecules, results also showed that ICD score was negatively correlated with immune activation genes, HLA molecule, chemokine receptors and chemokine genes. These results once again strongly prove our above conjecture. Therefore, we infer that the low ICD score group may have a good prognosis because of the high immune infiltration score, which suggests that the ICD score may have potential application in predicting immunotherapy response.

Studies have shown that high TMB can be used as an effective predictor of immunotherapy response, that is, high TMB corresponds to a good immunotherapy response.^39^ Therefore, we firstly classified all patients into high or low TMB groups in TNBC cohort then conducted differential analysis. The results indicated that high TMB group corresponded to lower ICD score, and TMB was negatively correlated with ICD score. This suggests that the low ICD score group may be more suitable for immunotherapy. To further confirm this, we conducted an analysis in an independent immunotherapy cohort in which patients all received ICIs therapy. Targeted ICIs therapy has ushered in a new era of cancer treatment.^52^ ICIs have been widely used in many other cancers with satisfactory results, including melanoma,^53^ colorectal cancer,^54^ lung cancer^55^ and so on. The IMpassion 130 trial provides unprecedented insights into breast cancer immunotherapy, making PD‐L1 a mature biological target in TNBC treatment.^56^ Our results showed patients in low ICD score group had an obviously survival advantage and higher immunotherapy remission rate, suggesting a significant immunotherapy response. Although there are currently some immunotherapy drugs recognized for use in TNBC patients, there is no data set on TNBC patients receiving immunotherapy in the existing public databases. We therefore selected the IMvigor210 cohort including 348 urothelial carcinoma patients who received immunotherapy, which is the most widely used cohort of similar studies.^57^, ^58^, ^59^ In the future, more research on TNBC immunotherapy responses and updates of related public databases will help to improve this issue. In addition, low ICD score group also had lower IC50 in some chemotherapy‐targeted drugs, that is, higher drug sensitivity.

At present, due to the lack of widely recognized targeted TNBC drugs in clinical treatment, patients still rely on systemic chemotherapy, resulting in inevitable side effects, resulting in poor treatment effect and high mortality.^60^ Activation of ICD to produce anti‐tumor effect is likely to be an effective way to treat TNBC in the future. In order to obtain the long‐term anti‐tumor immunity caused by ICD, ICD inducers have been continuously explored worldwide, and many new methods have been proved to have the ability to induce the occurrence of ICD.^61^ Our choice to validate DDX58(RIG‐I) and TREM1 is also based on this consideration. DDX58 can initiate an immune response to resist viral infections, while TREM1 can enhance non‐specific immune responses,^62^, ^63^ which means they are valuable targets in future immunotherapy research. Triggering the ICD with specific drugs, restoring the immunogenicity of “cold tumors”, and remodeling the TME for a greater antitumor immune response by exploiting innate and adaptive components would be a promising antitumor strategy.^64^, ^65^, ^66^ Mechanistically, when ICD occurs, DAMPs are exposed and released to interact with the pattern recognition receptor expressed by innate immune cells, resulting in the activation and maturation of these immune cells. They move to the lymph nodes to obtain sufficient antigen‐specific volume. When cancer antigens are subsequently exposed to T cells, immune cells will enter the tumor microenvironment to enhance immune infiltration.^40^, ^67^ Recently, a study reports a nanoparticle that precisely targets TNBC tumor tissue and converts intratumor bacteria into immune enhancers, thereby significantly enhancing the effect of TNBC immunotherapy.^68^ This study greatly demonstrates the feasibility of trigging the immune system in future immunotherapy and opens up more possibilities for the choice.

The main innovation of this study is that we combined TNBC with ICD, a unique immune‐related programmed cell death method, focused on immunotherapy, a highly potential treatment method, and created an ICD score that is very valuable. The immune correlation of this score has been verified from multiple perspectives based on different public databases, which will be very helpful in screening TNBC patients suitable for immunotherapy so that they can obtain the greatest therapeutic benefit. We also noticed some similar studies, such as Li's study,^69^ although they also focused on ICD and immunotherapy, they only set the sample to all breast cancers without distinguishing TNBC and the databases they selected are not as rich as ours. For another example, in Cheng's study,^70^ although they focused on TNBC and created a similar score for predicting immunotherapy, their validation of the immune correlation of the immune score was far less comprehensive than ours.

There still existed some limitations in our research. First, we only preliminary explored the role of ICD in TNBC. To deepen our understanding of the direct effects of ICD in TNBC, further in vitro and in vivo experimental validations are required to elucidate the specific molecular mechanisms and biological functions. Second, a larger sample size would improve the reliability of our findings.

## 5 CONCLUSION

The focus of our study is to elucidate the link between ICD subtypes and changes in TME of TNBC. These results may be beneficial for patients with TNBC to receive immunotherapy‐based interventions. We also formed and validated individualized ICD risk scores, which can be used as an indicator to access TME infiltration, reflect the general landscape of TME, forecast immunotherapy outcomes in TNBC patients, screen for appropriate immunotherapy candidates and conduct more targeted treatments. This research provides unique insights for individualize immune treatment strategies and promising immunotherapy candidates screening.

## AUTHOR CONTRIBUTIONS

**Jiachen Li:** Data curation (lead); formal analysis (lead); methodology (lead); software (lead); supervision (lead); validation (lead); visualization (lead); writing – original draft (lead); writing – review and editing (lead). **Zhengtian Li:** Data curation (lead); methodology (lead); project administration (equal); software (lead); validation (lead); visualization (lead); writing – original draft (equal); writing – review and editing (equal). **Wenkang Yang:** Methodology (lead); software (equal); visualization (equal); writing – original draft (lead); writing – review and editing (lead). **Jianmin Pan:** Data curation (equal); formal analysis (equal); methodology (equal); writing – original draft (equal); writing – review and editing (equal). **Huazong You:** Data curation (supporting); methodology (supporting); writing – original draft (supporting); writing – review and editing (supporting). **Lixiang Yang:** Data curation (equal); methodology (supporting); writing – original draft (supporting); writing – review and editing (supporting). **Xiaodong Zhang:** Conceptualization (lead); funding acquisition (lead); methodology (lead); project administration (lead); resources (equal); supervision (lead).

## FUNDING INFORMATION

This research was sponsored by the National Natural Science Foundation of China (grant no. 81760476).

## CONFLICT OF INTEREST STATEMENT

The authors have stated explicitly that there are no conflicts of interest in connection with this article.

## ETHICS STATEMENT

TCGA and GEO belong to public datasets. The patients involved in the databases have obtained ethical approval. Users can download relevant data for free to conduct research and publish relevant articles. This research has been approved by the Ethics Committee of the First Affiliated Hospital of Guangxi Medical University (NO. 2022‐E402‐01). All the procedures were performed in accordance with the relevant guidelines and regulations.

## Supporting information


**Figure S1.** GO and KEGG pathway analyses for the ICD phenotype‐related genes (DEGs) differentially expressed between different clusters. (A) The dot plot of top 10 GO annotations. (B) The dot plot of top 30 KEGG enrichments. The X‐axis represents the number of genes, the Y‐axis represents the GO or KEGG functional categories.

**Table S1.** ICD related genes.

## Data Availability

The raw data of this study are derived from the TCGA (https://portal.gdc.cancer.gov/) and GEO (https://www.ncbi.nlm.nih.gov/geo/), which are publicly available databases.
