# Supplementary figures and images for: Development and verification of a novel immunogenic cell death‐related signature for predicting the prognosis and immune infiltration in triple‐negative breast cancer
Source: Cancer Rep (Hoboken). 2024 Mar 1;7(3):e2007. doi: 10.1002/cnr2.2007 (PMC10905160; doi:10.1002/cnr2.2007)

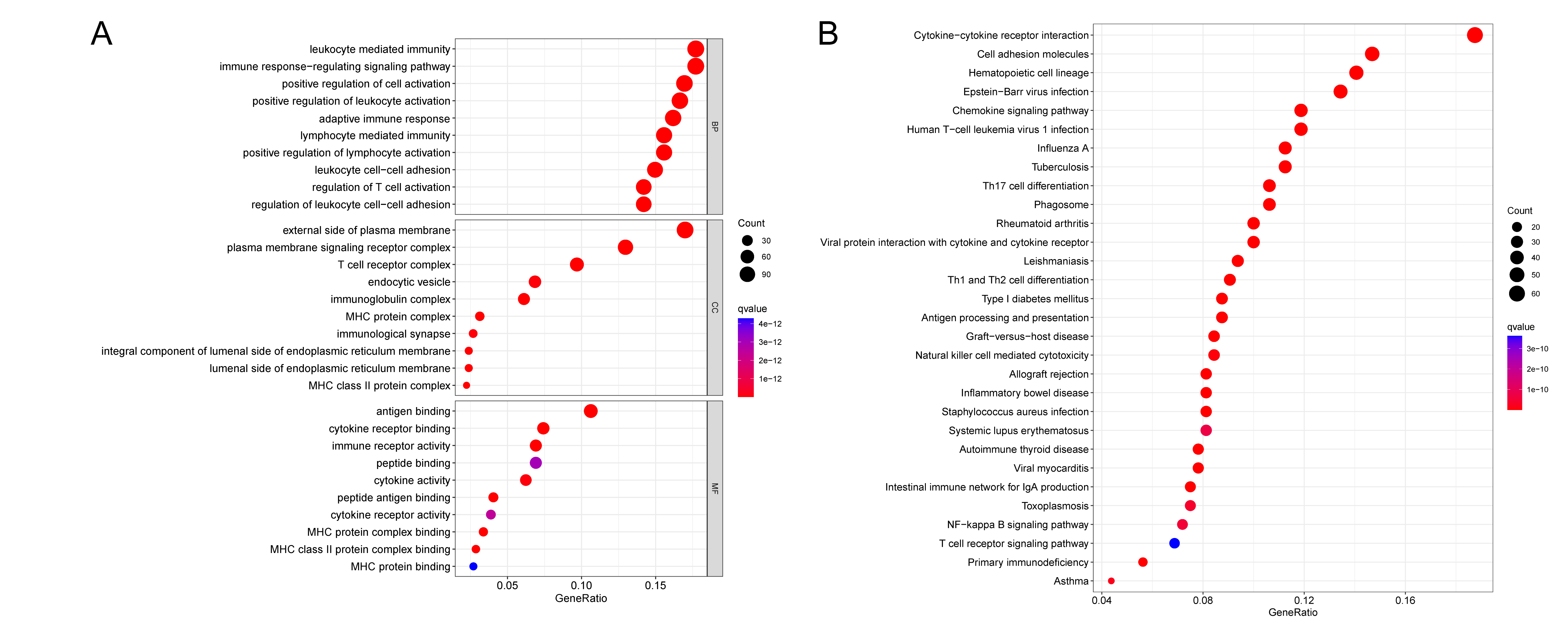

Supplement: Supplementary file 1 — Figure S1. GO and KEGG pathway analyses for the ICD phenotype‐related genes (DEGs) differentially expressed between different clusters. (A) The dot plot of top 10 GO annotations. (B) The dot plot of top 30 KEGG enrichments. The X‐axis represents the number of genes, the Y‐axis represents the GO or KEGG functional categories. [file CNR2-7-e2007-s001.tif]
