# Supplementary material for: Development and verification of a novel immunogenic cell death‐related signature for predicting the prognosis and immune infiltration in triple‐negative breast cancer
Source: Cancer Rep (Hoboken). 2024 Mar 1;7(3):e2007. doi: 10.1002/cnr2.2007 (PMC10905160; doi:10.1002/cnr2.2007)
Supplement: Supplementary file 2 — Table S1. ICD related genes. [file CNR2-7-e2007-s002.docx]

**Specific ICD-related genes**

| ID |  |  |  |
| --- | --- | --- | --- |
| CALR | DAMPs | Endoplasmic reticulum | Tumor cell uptake by DCs and chemotherapy-induced antitumoral immune response |
| HMGB1 | DAMPs | Translocates from the nucleus to the cytoplasm upon autophagy stimulation | Promotes the maturation and cross-presentation activity of APCs |
| HMGN1 | DAMPs | Cell nucleus and cytoplasm | Induces dendritic cell maturation, recruitment of APCs and antigen-specific immune responses |
| IL1A | DAMPs | Mesothelial cells | Cell activation, cytokine release |
| IL33 | DAMPs | Intracellular | Can bind ST2 on mast cells and TH2 cells and trigger secretion of pro-inflammatory and TH2 cyokines. The immunostimulatory activity of IL-33 might be inactivated during apoptosis |
| ROCK1 | DAMPs | Cytoplasm | Release the find-me signals ATP and UTP |
| PANX1 | DAMPs | Cell membrane | Release the find-me signals ATP and UTP |
| BCL2 | DAMPs | Nucleus membrane | Reduces reperfusion injury of skeletal or cardiac muscle when injected extracellularly |
| PPIA | DAMPs | Intracellular | Initiate and perpetuate the inflammatory response |
| HSPA4 | DAMPs | Cytoplasm | Protein folding, protein refolding, protein transport, and protein targeting |
| HSP90AA1 | DAMPs | Cytoplasm | Provides chaperoning activity for client proteins |
| TLR2 | Receptor (TLRs) | Ubiquitous, high in DCs, monocytes, macrophages and neutrophils | Promotes the production of pro-inflammatory cytokines and chemokines |
| TLR3 | Receptor (TLRs) | Ubiquitous, high in DCs, monocytes, macrophages and NK cells | Promotes the production of pro-inflammatory cytokines, chemokines and IFN-I |
| TLR4 | Receptor (TLRs) | Ubiquitous, high in DCs, monocytes, macrophages, neutrophils and endothelial cells | Promotes the production of pro-inflammatory cytokines, chemokines and IFN-I |
| TLR7 | Receptor (TLRs) | Ubiquitous, high in pDCs, monocytes, macrophages and B cells | Promotes the production of IFN-I and other cytokines and chemokines |
| TLR9 | Receptor (TLRs) | Ubiquitous, high in pDCs, monocytes, macrophages and B cells | Promotes the production of IFN-I and other cytokines and chemokines |
| CLEC4E | Receptor | Monocytes, macrophages, DCs, neutrophils and B cells | Promotes the release of pro-inflammatory cytokines |
| CLEC7A | Receptor | Monocytes, macrophages, DCs, neutrophils, mast cells, T and B cells | Initiating of intracellular signalling that produce pro-inflammatory cytokines |
| NLRP3 | Receptor (NLRs) | DCs, neutrophils, monocytes and macrophages | Promotes IL-1 and IL-18 secretion and initiates pyroptosis |
| DDX58 | Receptor | Ubiquitous, highly expressed in epithelial cells and myeloid cells | Trigger a transduction cascade which inducting the expression of antiviral cytokines |
| IFIH1 | Receptor | Cytoplasm, nucleus | Promotes the production of IFN-I and other cytokines and chemokines |
| CGAS | Receptor (CDSs) | Ubiquitous, highly expressed in epithelial cells, DCs, monocytes, macrophages and T cells | Promotes the production of IFN-I and other cytokines and chemokines |
| AIM2 | Receptor (CDSs) | Ubiquitous, highly expressed in epithelial cells, DCs, monocytes, macrophages, B cells and NK cells | Promotes IL-1 and IL-18 secretion and initiates pyroptosis |
| AGER | Receptor | Ubiquitous | Promotes the expression of pro-inflammatory genes, as well as cell migration, proliferation and apoptosis |
| TREM1 | Receptor (TREMs) | Myeloid cells, epithelial cells, endothelial cells and fibroblasts | Promotes pro-inflammatory cytokine and chemokine secretion |
| FPR1 | Receptor (GPCRs) | Ubiquitous, high in neutrophils, monocytes and macrophages | Promotes chemotaxis of neutrophils and monocytes/macrophages |
| FPR2 | Receptor (GPCRs) | Ubiquitous, high in neutrophils, monocytes and macrophages | Promotes chemotaxis of neutrophils and monocytes/macrophages |
| P2Y2R | Receptor (GPCRs) | Ubiquitous, high in epithelial cells, neutrophils, DCs, monocytes and macrophages | Promotes migration and activation of various immune cells |
| P2Y6R | Receptor (GPCRs) | Ubiquitous, high in stromal cells, neutrophils, monocytes, macrophages and T cells | Promotes proliferation and cytokine and chemokine production in stromal cells |
| P2Y12R | Receptor (GPCRs) | Mainly in platelets, also in DCs, monocytes, macrophages and T cells | Promotes platelet activation and Th17 differentiation |
| CASR | Receptor (GPCRs) | Ubiquitously expressed | Promotes monocyte/macrophage recruitment and NLRP3 activation |
| P2RX7 | Receptor (Ion channels) | Ubiquitous | Promotes cytokine and chemokine production, NLRP3 inflammasome activation and T cell activation |
